# Expansion of the Phenotypic Spectrum of TNRC6B-Related Neurodevelopmental Disorder in a Three-Generation Family with 22q13.1 Deletion

**DOI:** 10.3390/genes17040464

**Published:** 2026-04-15

**Authors:** Jessica Archer, Sheridan O’Donnell, Melissa Buckman, Nicole Bain, Himanshu Goel

**Affiliations:** 1 Hunter Genetics, New South Wales Health, Cnr. Turton Rd and Tinonee Rd., Waratah, NSW 2298, Australia; 2 Tamworth Community Health Centre, Tamworth, NSW 2340, Australia; 3 Department of Molecular Medicine, New South Wales Health Pathology, John Hunter Hospital, New Lambton Heights, NSW 2305, Australia; 4 School of Medicine and Public Health, University of Newcastle, Callaghan, NSW 2308, Australia

**Keywords:** *TNRC6B*, neurodevelopmental disorder, intellectual disability, developmental delay, copy-number variant (CNV), mRNA

## Abstract

**Background:** *TNRC6B* encodes a core effector of the RNA-induced silencing complex and is essential for miRNA-mediated gene silencing. Pathogenic variants in *TNRC6B* have recently been associated with a neurodevelopmental disorder characterised by developmental delay, intellectual disability, and behavioural difficulties. **Methods:** We report a three-generation family with a 22q13.1 deletion encompassing only exons 2–23 of *TNRC6B*. Clinical data were collected from medical records and family interviews, and the findings were compared with those of published cohorts. **Results:** Affected individuals presented with developmental delay, speech and language impairment, autism spectrum disorder, ADHD, oppositional defiant disorder, craniosynostosis, joint laxity, clinodactyly, and cardiac valve anomalies. The father and paternal grandmother had learning difficulties and neurobehavioral features, while the proband exhibited a more severe phenotype. **Conclusions:** This report expands the phenotypic spectrum of *TNRC6B*-related neurodevelopmental disorder, highlighting craniosynostosis, joint and connective tissue features, and cardiac involvement. Our findings also underscore variable expressivity across generations and emphasise the relevance of both copy-number and sequence variants in *TNRC6B* in patients with neurodevelopmental disorders.

## 1. Introduction

Precise post-transcriptional regulation of gene expression is essential for normal neurodevelopment. One principal mechanism controlling mRNA stability and translation is RNA silencing, a conserved process that adjusts protein production in response to developmental and cellular signals. Disruption of this pathway has been increasingly implicated in neurodevelopmental disorders [1]. RNA silencing regulates gene expression through small RNAs, including microRNAs (miRNAs). miRNAs are transcribed as primary transcripts, processed by Drosha–DGCR8 in the nucleus and by Dicer in the cytoplasm, and then loaded onto Argonaute (AGO1–AGO4) proteins to form the RNA-induced silencing complex (RISC) [2]. Within RISC, AGO proteins mediate mRNA cleavage or translational repression [3].

*TNRC6B* is located on chromosome 22q13.1 and comprises 23 exons encoding a large cytoplasmic protein of approximately 1833 amino acids (~194 kDa). It belongs to the GW182 family of scaffolding proteins (TNRC6A–C) that mediate miRNA-guided gene silencing. The N-terminal region, encoded by the early exons, is rich in glycine–tryptophan (GW) repeats. These provide docking sites for AGO proteins and form the core of the RISC. The central portion of the protein, encoded by the middle exons, contains a glutamine-rich region and a ubiquitin-associated (UBA) domain. These domains contribute to localisation within cytoplasmic processing bodies (P-bodies) and facilitate interaction with downstream effector complexes. The C-terminal region, encoded by the terminal exons, harbours an RNA recognition motif (RRM) and additional GW-rich sequences that directly bind RNA and recruit the cellular machinery responsible for mRNA deadenylation, decay, or translational repression [4,5,6,7].

The clinical significance of *TNRC6B* has only recently been established. A de novo frameshift and a de novo nonsense variant in *TNRC6B* were first noted in the Deciphering Developmental Disorders (DDD) study of whole exome sequencing for more than 2500 simplex families with a child with an autism spectrum disorder [8]. Granadillo et al.’s 2020 paper [9] described 17 individuals (12 males, 5 females) with heterozygous *TNRC6B* variants, including 7 nonsense, 5 frameshift, 2 splice-site, 2 intragenic deletions, and 1 missense variant. All individuals demonstrated developmental and neurobehavioral abnormalities, with high frequencies of speech delay (94%), autism or autistic traits (76%), ADHD (65%), hypotonia (59%), and variable skeletal and cardiovascular anomalies (see Table 1) [9]. Most prior studies have focused on single-nucleotide variants, whereas copy-number variants (CNVs) affecting *TNRC6B* remain underrepresented. Here, we describe a three-generation family with a 22q13.1 deletion encompassing only exons 2–23 of *TNRC6B*. Affected members demonstrated neurodevelopmental and behavioural features consistent with prior reports, along with previously under-recognised phenotypes, including craniosynostosis, joint laxity, clinodactyly, and cardiac valve anomalies. This study, therefore, expands the phenotypic spectrum of *TNRC6B*-related disorders and highlights variable intergenerational expression.

## 2. Methods

### 2.1. Genetic Testing

Chromosomal microarray analysis (CMA) was performed on the proband using Infinium CytoSNP-850K v1.4 (Illumina, San Diego, CA, USA) and data was analysed using BlueFuse Multi v4.5. Variants are reported using Genome Reference Assembly GRCh38 (hg38). A 22q13.1 deletion (chr22:40233976–40489019, GRCh38) encompassing *TNRC6B* (exon 2–23) was identified. No other gene was included in the deleted region. Segregation studies were performed in available family members via CMA.

### 2.2. Clinical Assessment

Clinical phenotyping included review of medical records, developmental assessments, and structured interviews with parents. Data collected encompassed growth parameters, developmental milestones, behavioural profiles, congenital anomalies, and imaging studies (brain MRI, echocardiogram). Standardised developmental scales, including Bayley Scales of Infant Development and Wechsler assessments, were incorporated when available.

### 2.3. Literature Review

A targeted literature search was performed in PubMed using “*TNRC6B*,” “22q13.1,” “neurodevelopmental disorder,” “intellectual disability,” and “RNA-induced silencing complex.” Phenotypic comparisons were made between published cases and the present family.

### 2.4. Ethical Considerations

Informed consent for genetic testing and publication of clinical data was obtained from the proband’s parents. This study was approved by the Hunter New England Research Office Ethics Manager; Reference: 20260116-001.

## 3. Case Report

### 3.1. Proband (IV-3; See Figure 1)

The proband was first reviewed at 7 months of age for global developmental delay and failure to thrive. She is the youngest of three sisters. She was born at 39 weeks after an unremarkable pregnancy via an elective Caesarean section. Her Apgar scores were 6 at 1 min, 7 at 5 min and 6 at 10 min, respectively. She required 21 h of respiratory support. She was treated for congenital pneumonia with 5 days of penicillin. Her birth weight was 2736 gm (5–10th percentile; 1.55 standard deviations below the mean for gestation), her length was 45 cm and her head circumference was 33 cm. She was re-admitted at 6 weeks of age for failure to thrive in the context of loose stools and frequent vomiting, subsequently managed with a proton pump inhibitor and feed thickener. Ultrasounds of her head and abdomen, including liver and kidneys, were normal. She did not have any other imaging.

**Figure 1:**
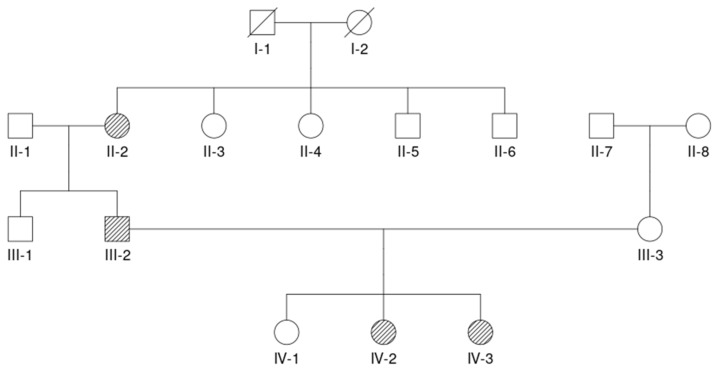
Family pedigree.

**Figure 2:**
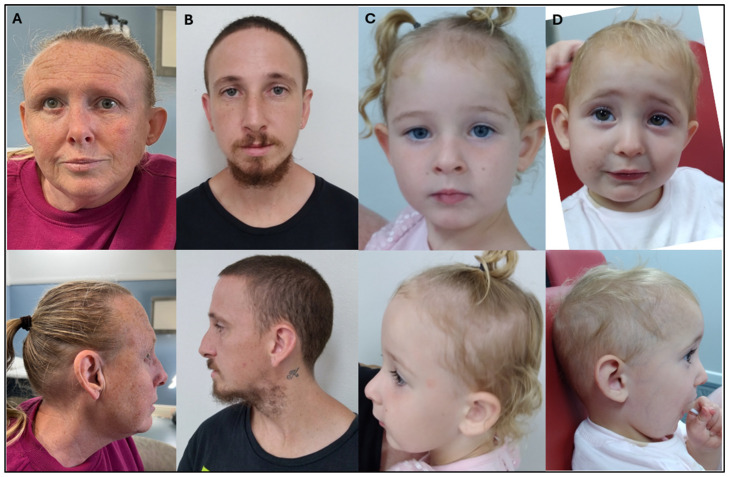
Clinical photography of the paternal grandmother (II-2; (**A**)), father (III-2; (**B**)), proband’s sister (IV-2; (**C**)), and proband (IV-3; (**D**)), demonstrating common features including downslanting palpebral fissures, bilateral epicanthi, hypoplastic nasal alae and micrognathia.

At 7 months of age, she was not sitting independently and was not crawling or babbling. At 13 months old, she was crawling, standing and cruising along furniture, waving, clapping and using a pincer grip. However, she only had two inconsistent words, did not produce varied vocalisations and primarily growled. She demonstrated head-banging behaviour, which was thought to be a form of sensory stimulation. She received a diagnosis of global developmental delay. At 16 months of age, her weight was at the 7th percentile and her head circumference was at the 9th percentile. She had downslanting palpebral fissures, bilateral epicanthi, hypoplastic nasal alae and micrognathia. (Figure 2) Joint laxity was felt to be greater than expected for her age. Her SNP microarray showed a deletion of 0.25 Mb in the chromosome 22q13.1 region.

### 3.2. Sister (IV-2)

The proband’s 3-year-old sister is being investigated for neurodevelopmental concerns. Her early developmental milestones were achieved within acceptable limits. At 3 years old, she could communicate in 3–4 word sentences and was toilet-trained during the daytime. She has ongoing early educational supports in place due to this genetic finding and her early education teachers noted an inability to socialise with other children. She is awaiting a formal assessment with an Autism Diagnostic Observation Schedule-trained provider. She exhibits head-banging when dysregulated.

She also has the same chromosome 22q13.1 deletion of 0.25 Mb. She was born at 39 weeks after a normal pregnancy via elective Caesarean section. Her APGAR scores were normal. Her birth parameters were within normal limits. Her joint laxity was milder than her sister. She has had episodes of recurrent vomiting followed by floppiness.

### 3.3. Sister (IV-1)

The proband’s 4-year-old sister has consistently achieved normal developmental milestones. She does not have the 22q13.1 deletion.

### 3.4. Father (III-2)

The proband’s 28-year-old father experienced neurodevelopmental challenges and he struggled academically compared to his unaffected brother (III-1). He obtained a full-scale IQ of 75 on WPSSI-R testing when he was 5 years of age, and a full-scale IQ of 83 on the WISC-III at 7 years of age. He had childhood diagnoses of Attention Deficit Hyperactivity Disorder (ADHD) and Oppositional Defiant Disorder (ODD), with ongoing behavioural dysregulation into adolescence. He underwent assessment for autism spectrum disorder (ASD) using the Autism Diagnostic Observation Schedule (ADOS) but was found not to meet criteria. He was born at 38 weeks of gestation with a birth weight of 2300 g (two standard deviations below the mean for gestation). He required a 5 day admission for nasogastric tube feeding support due to a poorly developed suck. Additional features included thin blonde hair, joint laxity, and mitral valve prolapse. He had a broad nose, a small, narrow chin and prominent ears (Figure 2). CMA confirmed the same 22q13.1 deletion.

### 3.5. Paternal Grandmother (II-2)

She is 52 years old and required surgery for craniosynostosis during childhood. There are no other members of her extended family that have craniosynostosis. She had strabismus and had multiple tympanostomy tube insertions for repeated ear infections. By early childhood, she had conductive hearing loss that was thought secondary to tympanic membrane damage. She wears hearing aids.

A computed tomograph (CT) scan of her brain demonstrated hyperostosis frontalis but no abnormalities of the brain. She has had bilateral inguinal hernia requiring surgical repairs and has a sub 1 cm hiatus hernia.

II-2 had learning difficulties and required additional educational support for maths and English. She did not complete secondary schooling. Despite government-supported initiatives funding her initial training, she was unable to successfully complete probationary periods in office-based secretarial jobs. However, in later adulthood she did complete a 6-week course that enabled her to work as a shop assistant in a pharmacy for two years. She first began using two-word combinations at 4 years of age. She was given a diagnosis of dyslexia in Grade 3. She has not undergone formal cognitive testing.

She exhibited clinodactyly and mild joint laxity, particularly of the ankles. She had a normal transthoracic echocardiogram in her 40s. Genetic testing confirmed the 22q13.1 deletion. She also has type 2 diabetes mellitus, paroxysmal atrial fibrillation, calcium oxalate renal stones, fibromyalgia and depression, which are not thought to be related to her genetic diagnosis.

Her husband (II-1) was unaffected. Her other son (III-1) declined genetics review but was placed in advanced classes at secondary school and has had a successful career working for the railway. His family do not think he is affected.

II-2’s siblings all performed well at school and have had successful careers. Efforts are ongoing to contact II-2’s siblings for segregation purposes.

## 4. Discussion

*TNRC6B* haploinsufficiency causes a distinct neurodevelopmental disorder characterised by developmental delay, intellectual disability (ID), speech impairment, ASD and ADHD. The present family demonstrates multigenerational segregation of a 22q13.1 deletion including only exons 2–23 of *TNRC6B*, with variable phenotypic expressivity, intergenerational phenotypic differences, and potentially under-recognised systemic features.

While core neurodevelopmental features were consistent, additional findings, including craniosynostosis, joint laxity, clinodactyly, and cardiac valve anomalies, represent potential associations with *TNRC6B* haploinsufficiency. The proband presented with developmental delay, speech impairment, and sleep disturbances, while the father and paternal grandmother exhibited learning difficulties and behavioural dysregulation (ADHD, ODD), reflecting variable severity across generations [7]. Behavioural phenotypes, particularly ASD, ADHD, and oppositional behaviours, are prominent in both published cases and the current family. Dysregulated miRNA networks have been implicated in ASD pathogenesis, targeting genes involved in synaptic function, metabolism, and immune response [17]. Although *TNRC6B*-associated syndromes generally present with mild dysmorphic features, our family illustrates expanded systemic involvement. Notably, cardiac involvement was evident in the father (floppy heart valve) and has been reported in previous cohorts as aortic root dilation in two patients [9]. While relatively uncommon, these findings suggest that *TNRC6B* may influence vascular or valvular development, potentially through miRNA-mediated regulation of developmental genes. These observations warrant longitudinal cardiovascular monitoring in affected individuals. Exome sequencing of 362 probands with non-syndromic tetralogy of Fallot (TOF) and their parents within the Paediatric Cardiac Genomics Consortium (PCGC) showed one individual with a de novo heterozygous c.2482C>T variant (p.Gln828*) [18]. Our family’s CNV (22q13.1 deletion encompassing only exons 2–23 of *TNRC6B*) further underscores that haploinsufficiency, whether by CNV or sequence variant, produces overlapping phenotypes. Granadillo et al. [9] described 17 individuals with heterozygous *TNRC6B* variants (see Table 1). All individuals demonstrated developmental and neurobehavioral abnormalities, with high frequencies of speech delay (94%), autism or autistic traits (76%), ADHD (65%), hypotonia (59%), and variable skeletal and cardiovascular anomalies [9]. Joint hypermobility or other connective tissue-like features were observed in 8/17 in their cohort, which is supported by the finding of joint laxity in our family. Genotype–phenotype analysis suggested that N-terminal variants affecting the Argonaute-binding domain were associated with macrocephaly, whereas C-terminal variants affecting the silencing domain were linked to microcephaly. However, no strong correlations were observed with specific neurodevelopmental symptoms. Notably, 23% of pathogenic variants were inherited, emphasising the importance of genetic counselling and highlighting variable expression among carriers. Speech delay was the most prevalent developmental challenge, while autism and ADHD were frequently observed neurobehavioral traits. Loss-of-function variants in *TNRC6B;* c.2040G>A, p.(Trp680*) and c.830_836del, p.(Asn277Metfs*3) were reported in patients with developmental language disorders, like childhood apraxia of speech (CAS) [11,13]. Functional studies have also demonstrated that synonymous variants, such as c.3141G>A, can disrupt RNA splicing, highlighting the diverse molecular mechanisms by which *TNRC6B* variants can contribute to disease [19]. A 31-year-old woman with epilepsy with onset during infancy and ASD without ID had a de novo pathogenic variant in the *TNRC6B*: c.2189del, p.(Gln730Argfs*62) [12].

Mild dysmorphic features were noted previously, though no consistent facial pattern emerged. Two unrelated Chinese patients had de novo *TNRC6B* variants, c.335C>T (p.Pro112Leu) and c.1632delC (p.Leu546fs*63). The clinical features of the patients were DD/ID, delayed speech, ADHD, behavioural abnormalities, short stature, low body weight, café-au-lait spots, metabolic abnormalities, and facial dysmorphism, including coarse facial features, sparse hair, frontal bossing, hypertelorism, amblyopia, strabismus, and downslanting palpebral fissures [10].

*TNRC6B*-associated ID, ADHD, and autism share similarities with other RNA-induced silencing complex (RISC) disorders. Disease-causing variants in *AGO1* and *AGO2* have also been linked to ID and autism, while expansions of intronic TTTCA and TTTTA repeats in *TNRC6A* are implicated in benign familial myoclonic epilepsy [20,21,22,23]. *TNRC6B* bridges AGO-bound microRNAs to downstream effector complexes, including PAN2–PAN3 and CCR4–NOT, thereby enabling translational repression and deadenylation-dependent mRNA decay. This function is particularly relevant to neurodevelopment, as miRISC components localise to dendrites and P-body-like structures that regulate activity-dependent local translation near synapses. More broadly, *TNRC6B* dosage can be limiting for RNA-silencing output, supporting a dosage-sensitive model in which partial loss of *TNRC6B* disrupts finely balanced post-transcriptional regulation of neurodevelopmental gene networks. These mechanistic data are consistent with the human phenotype associated with heterozygous pathogenic *TNRC6B* variants, which includes developmental delay, speech impairment, autism spectrum disorder, ADHD, and related neurobehavioral features [24,25].

Marked intrafamilial variability is evident in *TNRC6B*-associated neurodevelopmental disorder, with phenotypes ranging from isolated learning difficulties and behavioural traits to global developmental delay and structural anomalies. Several non–mutually exclusive mechanisms may underlie this heterogeneity. Allelic heterogeneity and variant position are critical determinants: premature termination codons (PTCs) located upstream of the final exon–exon junction are predicted to trigger nonsense-mediated decay (NMD), resulting in haploinsufficiency. In contrast, truncating variants in the terminal exon may escape NMD and produce partially functional proteins, depending on preservation of key domains such as the AGO-binding GW repeats or the C-terminal RRM and silencing regions. In addition, although typically inefficient, basal stop-codon readthrough could theoretically permit low-level synthesis of full-length protein from certain PTC alleles, potentially modifying residual *TNRC6B* activity and contributing to phenotypic variability [26].

Beyond allele-specific effects, *TNRC6B* functions as a dosage-sensitive scaffold within the RNA-induced silencing complex (RISC), and modest differences in residual protein levels may disproportionately affect miRNA-regulated neurodevelopmental gene networks. Variable buffering by paralogues (TNRC6A and TNRC6C), together with genetic modifiers within the miRNA pathway, likely further shapes the clinical spectrum observed in *TNRC6B*-related disorder.

Limitations of this study include that no member of this family underwent additional genomic testing, such as exome sequencing. It was not clinically indicated in this context; however, this would have given further weight to the association between the phenotypes described in our family and *TNRC6B* haploinsufficiency. The same is true regarding II-2’s craniosynostosis. Sequencing of other genes with known associations with craniosynostosis would have provided further support for this proposed association between *TNRC6B* and craniosynostosis; however, it was not thought to be indicated clinically as craniosynostosis was not a priority for II-2. Interestingly, no other member of the extended family has craniosynostosis, which makes a classically described autosomal dominant craniosynostosis syndrome such as *TWIST1* or *FGFR1* less likely. Moreover, one of two brothers described in Babbs et al. (2014) with a chromosome 22 pericentric inversion that disrupted *TNRC6B* and *TCF20* had metopic and coronal synostosis, although they concluded that *TCF20* and not *TNRC6B* was responsible for the brothers’ phenotype [14].

## 5. Conclusions

This three-generation family confirms *TNRC6B* haploinsufficiency as a cause of neurodevelopmental disorder and expands the phenotypic spectrum to include craniosynostosis, connective tissue features, and cardiac anomalies, although further study is required. Variable expressivity across generations emphasises the need for careful genetic counselling. Recognition of both copy-number and sequence variants in *TNRC6B* is essential for accurate diagnosis, prognostication, and management of affected individuals. Further studies are warranted to clarify genotype–phenotype correlations and elucidate mechanisms underlying variable expression.

## Figures and Tables

**Table 1:** Clinical and molecular spectrum associated with *TNRC6B* variants across published cohorts and current cases.

Source	Sex	Age (Rounded to Nearest Full Year)	Developmental Delay/Intellectual Disability	IQ	ASD	ADHD	Other Behavioural Issues	Inheritance	Musculoskeletal/Connective Tissue Features	Other Congenital Malformations	Other Medical Problems
Current	F	1	present	Not available	Too young	Too young	Head banging	Paternal	Joint laxity	absent	absent
Current	F	3	absent	Not available	Suspected	unknown	Head banging	Paternal	Mild joint laxity	absent	absent
Current	M	28	present	WPSSIR: 75 WISCIII: 83	absent	present	ODD, behavioural dysregulation	Maternal	Joint laxity	Mitral valve prolapse, sparse hair	absent
Current	F	52	present	Not available	absent	absent	absent	Unknown (parents deceased)	Joint laxity, craniosynostosis	absent	Repeated ear infections resulting in hearing loss, bilateral inguinal hernias
Yang et al. 2024 [10]	F	7	present	51	absent	present	Irritability, poor concentration	De novo	absent	Abnormal brain MRI, sparse hair, abnormal EEG	absent
Yang et al. 2024 [10]	M	3	present	96	absent	absent	absent	De novo	absent	Café-au-lait macules, short stature	Metabolic abnormalities
Yahia et al. 2024 [11]	unknown	unknown	present	Not available	unknown	unknown	Behavioural abnormalities	De novo	unknown	unknown	unknown
Bellido-Cuellar et al. 2023 [12]	F	31	absent	WASI-II: 84/87/84	present	present	absent	De novo	Joint laxity, scoliosis	Abnormal EEG	absent
Granadillo et al. 2020 [9] #1	M	6	present	Not available	Poor socialisation	present	absent	Unknown	absent	PFO, dilated aortic root, RV conduction delay, Chiari type 1 malformation, congenital entropian	absent
Granadillo et al. 2020 [9] #2	M	6	present	Not available	present	present	absent	De novo	absent		
Granadillo et al. 2020 [9] #3	F	3.7	present	Not available	present	present	absent	De novo	absent	Right temporal arachnoid cyst	
Granadillo et al. 2020 [9] #4	F	11	present	IQ—73 DQ 76 Nonverbal 95	Autistic features	present	Anxiety, depression, behavioural difficulties	De novo	Hypermobility of elbows, slender fingers and build	Chiari Type 1 malformation, Bilateral SNHL	Chronic otitis media, intermittent staring spells with normal EEG
Granadillo et al. 2020 [9] #5	M	6	present	Not available	Autistic features	absent	Aggressiveness	Paternal	absent	Hydrocele, sacral dimple, abnormal aorta and abnormal origin of R coronary artery, microcephaly, bilateral SNHL	Inguinal hernia, GERD, feeding issues
Granadillo et al. 2020 [9] #6	M	15	present	97	present	present	ODD	Maternal	Small joint hypermobility	absent	absent
Granadillo et al. 2020 [9] #7	M	12	present	VIQ: 105 PIQ: 85	present	present	absent	De novo	Joint hypermobility	absent	Benign nocturnal alternating hemiplegia of childhood
Granadillo et al. 2020 [9] #8	M	12	present	63	Autistic features	present	absent	De novo	Sprengel anomaly	absent	SCAD deficiency
Granadillo et al. 2020 [9] #9	M	17	present	73	present	absent	absent	Unknown	Recurrent patella subluxation bilaterally	Left supernumerary nipple	absent
Granadillo et al. 2020 [9] #10	M	13	present	72	present	absent	absent	De novo	Pes planus, hyperpigmentary lesions on wrist and upper leg	Left supernumerary nipple	Recurrent ear infections
Granadillo et al. 2020 [9] #11	M	13	present	55	absent	absent	Impulsivity	De novo	Muscle weakness	Abnormal brain MRI	Hyperreflexia
Granadillo et al. 2020 [9] #12	M	2.6	present	53	present	absent	absent	De novo	absent	absent	Myoclonus epilepsy
Granadillo et al. 2020 [9] #13	M	14	present	WISC: VCI 79, PRI 71, PSI 73	present	present	absent	Maternal	Broad palms, tibial malformation	Cryptorchidism	absent
Granadillo et al. 2020 [9] #14	F	10	present	80	absent	present	absent	De novo	Clinodactyly	Imperforate anus, vestibular fistula	Central precocious puberty
Granadillo et al. 2020 [9] #15	F	11	present	50	absent	present	Anger with tremor	Unknown	Joint hypermobility	absent	Bilateral inguinal hernia
Granadillo et al. 2020 [9] #16	F	6	present	WASI-II FS-IQ 113, Verbal Comprehension 131	absent	present	Impulsivity	Unknown	Pes planus, scoliosis, muscle atrophy in legs	Conductive hearing loss	Constipation, recurrent ear infections
Granadillo et al. 2020 [9] #17	M	16	present	Not available	Autistic features	absent	absent	De novo	Marfanoid features. joint pain, long slender fingers	Wide aortic root	Swallowing difficulties
Eising et al. 2019 [13]	F	4	absent	“average”	present	unknown	unknown	Unknown	unknown	unknown	unknown
Iossifov et al. 2014 [8]	M	unknown	unknown	78	present	unknown	unknown	unknown	unknown	unknown	unknown
Iossifov et al. 2014 [8]	M	unknown	unknown	81	present	unknown	unknown	unknown	unknown	unknown	unknown
The following may have a second genetic variant contributing to phenotype											
Babbs et al. 2014 [14] *Pericentric inversion disrupting TCF20 and TNRC6B*	M	1	present	79	present	absent	absent	Presumed parental mosaicism	Metopic and coronal synostosis	absent	absent
Babbs et al. 2014 [14] *Pericentric inversion disrupting TCF20 and TNRC6B*	M	10	present	Moderate to severe on Vineland Adaptive Behavioural Scores	present	absent	absent	Presumed parental mosaicism	absent	absent	absent
Mitani et al. 2021 [15] *Also homozygous for ADSL: NM_000026.4; c.1277G>A (p.Arg426His)*	M	2	present	Not available	absent	present	absent	Homozygous for a missense variant in *TNRC6B*	absent	Abnormal brain MRI	absent
Mitani et al. 2021 [15] *Also homozygous for ADSL: NM_000026.4; c.1277G>A (p.Arg426His)*	F	10	present	Not available	absent	present	absent	Homozygous for a missense variant in *TNRC6B*	absent	absent	absent
Deng et al. 2025 [16] *Also had Xq28 intragenic microdeletion involving exons 2 and 3 of MECP2*	F	1.5	present	<1%ile on Griffith Mental Developmental Scales	absent	absent	absent	unknown	absent	absent	Short stature (z-score: 5.29), microcephaly

## Data Availability

Data supporting reported results available on request from corresponding author.

## Abbreviations

The following abbreviations are used in this manuscript:

RNA	Ribonucleic acid
miRNA	Micro-Ribonucleic acid
ADHD	Attention Deficit Hyperactivity Disorder
CNV	Copy-number variant
RISC	RNA-induced silencing complex
AGO	Argonaute
mRNA	Messenger Ribonucleic acid
GW	glycine–tryptophan
UBA	ubiquitin-associated
RRM	RNA recognition motif
DDD	Deciphering Developmental Disorders
CMA	Chromosomal microarray
MRI	Magnetic Resonance Imaging
SNP	Single Nucleotide Polymorphism
APGAR	Appearance, Pulse, Grimace, Activity, Respiration
IQ	Intelligence Quotient
WPSSI-R	Wechsler Preschool and Primary Scale of Intelligence—Revised
WISC-III	Wechsler Intelligence Scale for Children—Third Edition
ASD	autism spectrum disorder
ADOS	Autism Diagnostic Observation Schedule
ODD	Oppositional Defiant Disorder
CT	Computed Tomography
ID	Intellectual disability
TOF	tetralogy of Fallot
PCGC	Paediatric Cardiac Genomics Consortium
PTCs	premature termination codons
NMD	nonsense-mediated decay
